# Genesis of fecal floatation is causally linked to gut microbial colonization in mice

**DOI:** 10.1038/s41598-022-22626-x

**Published:** 2022-10-27

**Authors:** Syed Mohammed Musheer Aalam, Daphne Norma Crasta, Pooja Roy, A. Lee Miller, Scott I. Gamb, Stephen Johnson, Lisa M. Till, Jun Chen, Purna Kashyap, Nagarajan Kannan

**Affiliations:** 1 Division of Experimental Pathology, Department of Laboratory Medicine and Pathology, Mayo Clinic, 200 First St SW, Rochester, MN 55905 USA; 2 Department of Orthopedic Surgery, Mayo Clinic, Rochester, MN 55905 USA; 3 Microscopy and Cell Analysis Core, Mayo Clinic, Rochester, MN 55905 USA; 4 Division of Computational Biology, Department of Quantitative Health Sciences, Mayo Clinic, Rochester, MN 55905 USA; 5 Department of Gastroenterology, Mayo Clinic, Rochester, MN 55905 USA; 6 Center for Regenerative Biotherapeutics, Mayo Clinic, Rochester, MN 55905 USA; 7 Mayo Clinic Cancer Center, Mayo Clinic, Rochester, MN 55905 USA

**Keywords:** Gastrointestinal models, Microbiology, Gastroenterology

## Abstract

The origin of fecal floatation phenomenon remains poorly understood. Following our serendipitous discovery of differences in buoyancy of feces from germ-free and conventional mice, we characterized microbial and physical properties of feces from germ-free and gut-colonized (conventional and conventionalized) mice. The gut-colonization associated differences were assessed in feces using DNA, bacterial-PCR, scanning electron microscopy, FACS, thermogravimetry and pycnometry. Based on the differences in buoyancy of feces, we developed levô in fimo test (LIFT) to distinguish sinking feces (sinkers) of germ-free mice from floating feces (floaters) of gut-colonized mice. By simultaneous tracking of microbiota densities and gut colonization kinetics in fecal transplanted mice, we provide first direct evidence of causal relationship between gut microbial colonization and fecal floatation. Rare discordance in LIFT and microbiota density indicated that enrichment of gasogenic gut colonizers may be necessary for fecal floatation. Finally, fecal metagenomics analysis of ‘floaters’ from conventional and syngeneic fecal transplanted mice identified colonization of > 10 gasogenic bacterial species including highly prevalent *B. ovatus,* an anaerobic commensal bacteria linked with flatulence and intestinal bowel diseases. The findings reported here will improve our understanding of food microbial biotransformation and gut microbial regulators of fecal floatation in human health and disease.

## Introduction

Feces of more than ten percent of healthy individuals consistently float and this is not linked to any specific pathology^1^. Notably, one out of four patients with functional bowel disorder experience fecal floatation^2^. The mechanism of fecal floatation is not well understood. Experimental models to investigate factors influencing fecal floatation would be of general interest but are currently not available.

Feces offers a potential vantage point to study gut microbial dynamics in gut health since feces constitutes the largest reservoir of microorganisms in the order of 100 billion per gram^3^. Notably, germ-free mice have greatly enlarged cecums because of mucus and undigested fiber accumulation, and the small intestines are less developed, often leading to reproductive and functional gastrointestinal disorders^4,5^. It is estimated that combined dead and living bacteria contribute to a significant proportion (25–54%) of dry solids in feces^3,6^. In a healthy adult, the endogenous microbially produced gut gases (hydrogen, methane, and insignificant quantities of odorogenic sulfides and ammonia) are only a minor amount as opposed to bulk of gases, predominantly oxygen, nitrogen, and carbon dioxide, generated every day from exogenous air that enter GI tract during ingestion of food and water^7,8^. The colon is a major source of endogenous gases due to the higher density of microbiota that ferment foods containing poorly digested carbohydrates^21^. These fermented gases build up and are periodically evacuated as flatulence but also could be trapped in fecal biomass thereby reducing their specific gravity^1^. Therefore, the specific gravity of feces could be influenced by lifestyle, food, water and gas composition, and microbial diversity.

While investigating microbial colonization in a gnotobiotic mouse model, we serendipitously discovered distinct sinking and floating behaviors of feces from germ-free and conventional mice, respectively. Detailed analysis of the specific gravity measurements of standard food and fecal pellets from germ-free and gut-colonized mice indicated a significant reduction in the specific gravity of feces from gut-colonized mice. We exploited these distinctions to establish a new method, namely the levô in fimo test (LIFT) to distinguish sinking individual fecal pellets (sinkers) of germ-free mice from floating fecal pellets (floaters) of gut-colonized mice. By introducing gut microbial colonizers in germ-free mice using fecal or environmental transmission, we could physically alter fecal properties from ‘sinkers’ to ‘floaters’, thereby causally linking fecal floatation to gut microbial colonization in highly controlled laboratory mice fed on standard diet. The study demonstrates the utility of our model and methods to identify microbial species and other factors influencing fecal floatation and associated gut health.

## Results

In this study, we have characterized the properties of fecal pellets obtained from conventional C57BL/6 (B6) mice and their age-matched gnotobiotic mice bred in germ-free isolators (referred hereafter as germ-free mice). To investigate the causal relationship between fecal properties and gut microbial colonization, we collected feces from gnotobiotic mice exposed to single intragastric gavage of fecal microbiota preparation from either a single conventional donor mouse (hereinafter referred as mouse FMT) or two human donors (hereinafter referred as human FMT), or sterile PBS (hereinafter referred as environmental microbial exposure group or Env MT). Following intragastric gavage, cages were transferred to conventional housing facility. Except germ-free mice, all other mice are collectively referred to as gut-colonized mice. The fresh fecal pellets from all groups had the typical granular shape, size, and dark brown coloration indistinguishable from each other (data not shown).

### Superior gut colonization by syngeneic microbial transmission compared to human fecal or environmental microbial transmission in B6 mice

**Figure 1:**
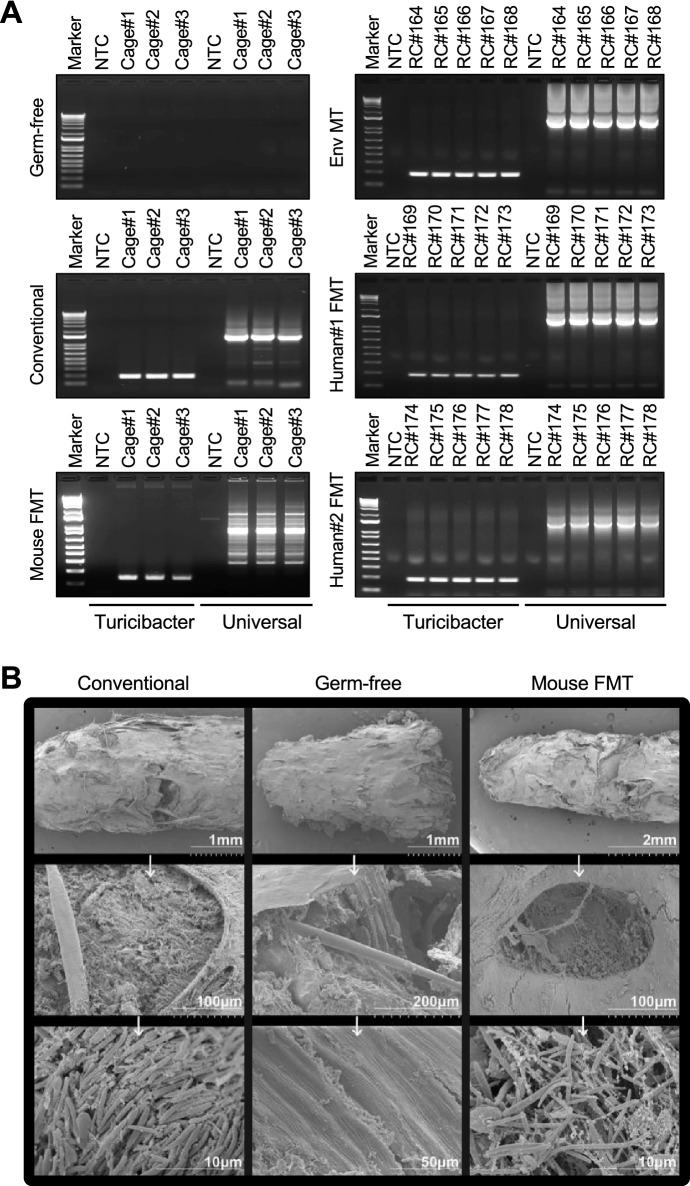
Scanning electron microscopy and PCR analysis of mouse feces. (**A**) PCR analysis of fecal DNA isolated from conventional, germ-free, and gut-colonized (Env MT, mouse FMT and human FMT) mice using bacterial universal and turicibacter primers. The absence of PCR amplicons is indicative of the lack of bacterial gut colonization in germ-free B6 mice. Detection of PCR amplicons in gut-colonized mice is suggestive of efficient gut microbial colonization at levels similar to that of conventional B6 mice. The samples were loaded in the sequential order and the unloaded wells were cropped from the image. RC# represents number given to individual animal. (**B)** Representative SEM images of fecal samples from germ-free, conventional and mouse FMT mice. Scale bars 10 µM—2 mm. No bacteria are found in the feces of germ-free mice. Different types of bacteria are found in the feces of conventional and mouse FMT mice.

**Figure 2:**
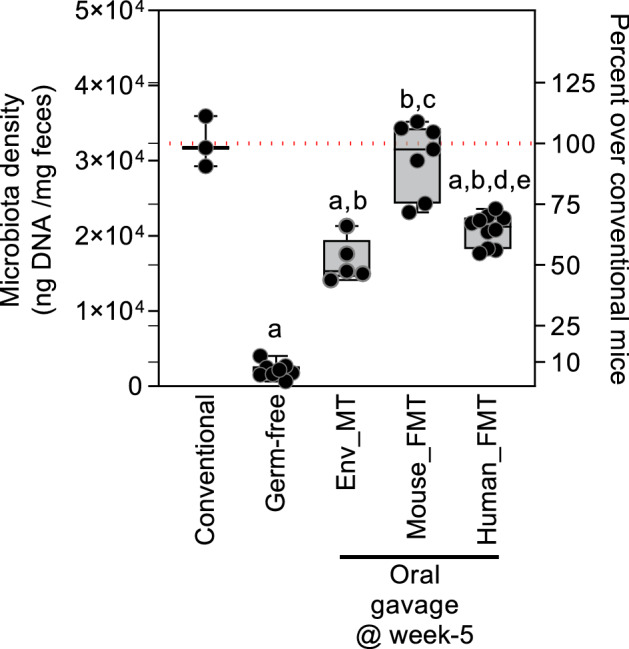
Variation in gut microbiota density in gut-colonized mice. Total fecal DNA levels per mg wet weight of feces. Ordinary one-way ANOVA was performed for comparisons between the samples. ^a^*p *< 0.0001 versus Conventional, ^b^*p *< 0.0001 versus Germ-free, ^c^*p *< 0.0001 versus Env MT, ^d^*p *< 0.05 versus Env MT and ^e^*p *< 0.0001 versus Mouse FMT.

To characterize the level of fecal microbial presence and density in mice, we performed PCR, scanning electron microscopy (EM), and DNA yield measurements. PCR confirmed complete absence of bacteria in all fecal pellets tested from 8-week old germ-free mice and the presence of bacteria in all other age-matched gut-colonized mice (Fig. 1A). Additionally, feces of germ-free mice were cultured in Sabouraud dextrose media, brain–heart infusion media, and nutrient broth media at 37 °C for 7 days under aerobic and anaerobic conditions. The absence of  microbial growth activity under these conditions indicated germ-free status of feces in our mice (data not shown). To further substantiate these observations, we examined the feces obtained from germ-free, conventional, and mouse FMT mice by scanning EM. In scanning EM, we observed diverse and dense microbial flora in fecal samples of conventional and mouse FMT mice, indicative of biomass transformation (Fig. 1B). As expected, scanning EM did not find any microorganisms in the feces of gnotobiotic germ-free mice^9^.To determine the fitness of gut microbial colonization we measured DNA content in fecal biomass to estimate gut microbiota density in all 5 groups of B6 mice^10^ . The conventional and mouse FMT mice showed similar DNA yields of 32 µg and 30 µg respectively, per mg wet weight of feces, while the DNA yield from human FMT and Env MT mice was around 21 µg and 16 µg respectively, per mg wet weight of feces (Fig. 2). This observation suggests that fecal microbiota from the same species show better microbiota densities and fitness in gut colonization than the cross-species fecal microbiota. Interestingly, the DNA yield from the feces of Env MT mice was half (~ 16.6 µg per mg wet weight of feces) that of conventional mice (Fig. 2). However, total fecal DNA yield in germ-free B6 mice was ~ 2–3 µg per mg wet weight of feces, reduction (< 10%) compared to conventional B6 mice (Fig. 2), and consistent with expected host cell genomic DNA presence in the feces^11^. These observations confirm that transmission of syngeneic microbiota in mice demonstrates higher gut colonization fitness compared to environmental or human fecal microbiota^12,13^.

### Flow cytometry identifies inverse association between fecal microbiota and food biomass transformation

**Figure 3:**
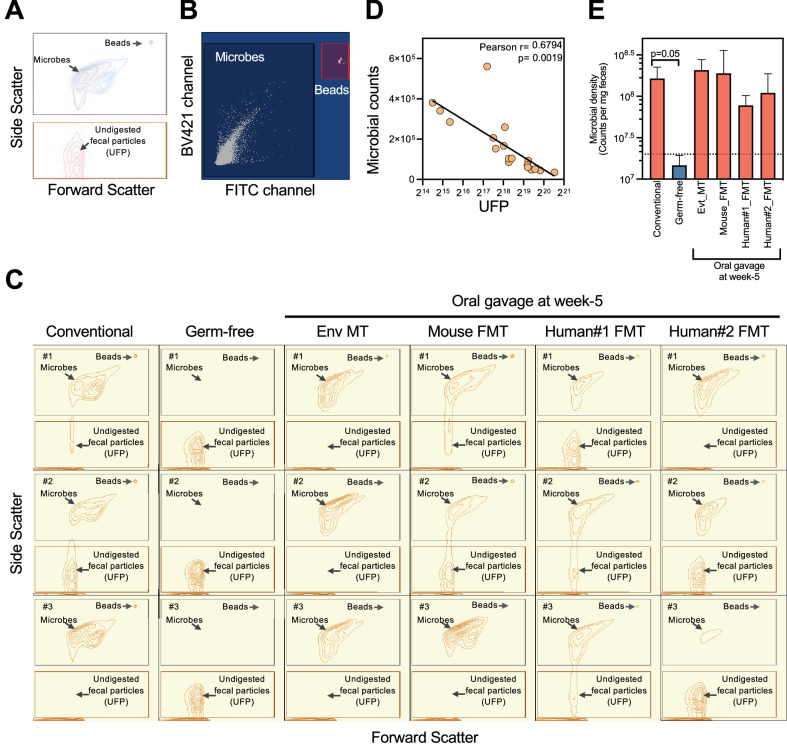
Quantitative flow cytometry analysis of fecal microbial dynamics. (**A**) Overlayed contour FACS plots of conventional and human FMT samples showing 2 distinct clusters of microbial flora in the upper half of the plot. The human FMT group shows presence of only 1 of the 2 clusters seen in the conventional mice samples. Arrows indicate microbial presence, UFP and beads. (**B)** Representation of gates drawn for microorganisms and counting beads using flow cytometry analysis. (**C**) Flow cytometry plots of 3 fecal samples from each group depicting lack of distinct microbial cluster and enrichment of fecal UFP in germ-free feces. These plots also highlight differing levels of microbial and UFP particles in feces. It is notable that even in germ-free feces, low background counts are consistently observed which may be due to particles introduced from feces and sample processing steps. (**D**) Pearson’s (non-linear regression) correlation between the fecal UFP and the fecal microbial counts for all fecal samples. The outlier in data could represent occasional disrupted relationship between the microbial density and UFP. (**E**) Absolute microbial counts per mg feces.

Feces is the solid organic refuse of a living organism, mainly composed of water, metabolic waste, and undigested materials, in addition to excreted microbiota and host cells^6^. To determine the microbiota densities and to test the fitness of fecal microbial transplants (FMT; mouse and human donors) in B6 mice, we developed a fluorescence-activated cell sorting (FACS) based strategy to distinguish fecal microbiota from fecal undigested food particles (UFP) based on size and granularity (Fig. 3A–E).

Further to quantify the microbiota densities per mg of feces, we spiked-in fecal suspensions with an internal standard of microsphere beads (Fig. 3B). This method allowed quantification of the microbiota densities with no further need to culture microorganisms. The analysis of age-matched germ-free and conventional B6 mice feces clearly distinguished microbiota from UFP and internal standard microsphere beads (Fig. 3C). However, inter-feces differences in microbiota densities and UFP were observed in conventional, mouse FMT, and human FMT samples (Fig. 3C). Interestingly, the Env MT group showed complete fecal biomass transformation to microbiota densities (Fig. 3C,E) like that of conventional or mouse FMT groups. However, feces from human FMT mice had lower microbial and higher UFP particles (Fig. 3C,E). We then analyzed the relationship between the proportion of UFP and microbiota. To our surprise, a significant inverse correlation between UFP and microbiota (*p *= 0.0019; r = 0.6794) (Fig. 3D,E) was observed, suggesting that gut passage of food in the presence or absence of gut colonizers may lead to compositionally different fecal biomass.

### Thermogravimetric analysis distinguishes feces of germ-free versus gut-colonized mice

**Figure 4:**
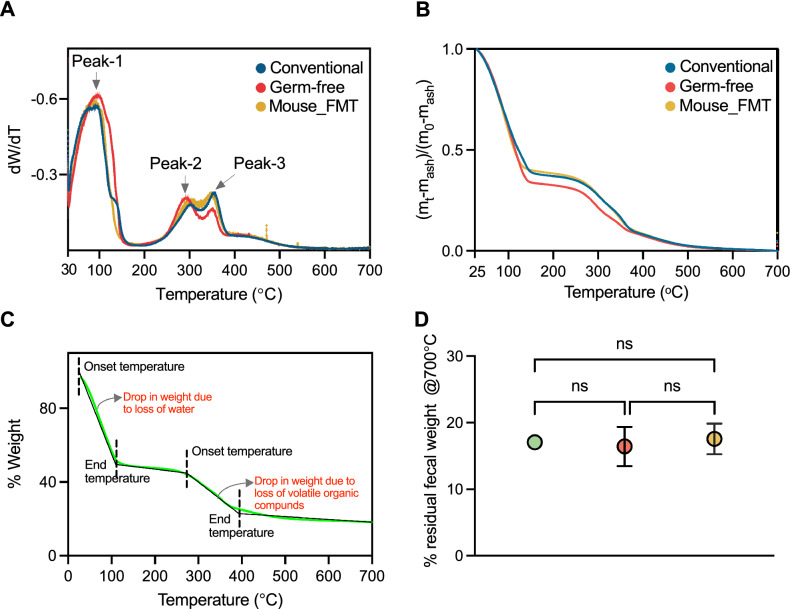
Thermogravimetric analysis (TGA) of fecal samples. (**A**) Representative plot showing decrease in percent weight with increase in temperature (°Celsius;  °C) during the TGA of fecal samples under pyrolysis conditions. (**B**) Effect of heating rate on the change of mass with increasing temperature for conventional, germ-free and mouse FMT fecal samples. Data is depicted in dry ash free basis. (**C**) Derivative thermogravimetric plot for weight loss against increasing temperatures for three groups. (**D**) Percent residue fecal weights obtained from standard TGA curve for three groups. Repeated measures one-way ANOVA was performed for comparisons between the samples and ns = not significant.

Fecal biomass compositional differences are grossly analyzed by pyrolysis, a thermochemical process that involves the decomposition of organic materials at elevated temperatures in the absence of oxygen or in an atmosphere of inert gases^14^. Thermogravimetric (TGA) analysis during pyrolysis allows the determination of change in mass of an organic substance over a range of operating temperatures (Fig. 4A). Therefore, to determine the change in mass of the feces as a function of its organic content, we performed TGA analysis on fresh fecal samples obtained from germ-free, conventional, and mouse FMT groups. Interestingly, we observed that the change in fecal mass of conventional and mouse FMT was overall identical but distinct from that of germ-free fecal mass at least over three different temperature ranges (Fig. 4B–C). Further, the analysis indicated that feces from germ-free mice required a lower temperature for pyrolysis relative to feces from conventional or mouse FMT groups (Fig. 4C). However, we did not observe any difference in residual fecal ash mass between germ-free, conventional, and mouse FMT groups (Fig. 4D).

### Specific gravity distinguishes feces in germ-free versus gut-colonized mice

**Figure 5:**
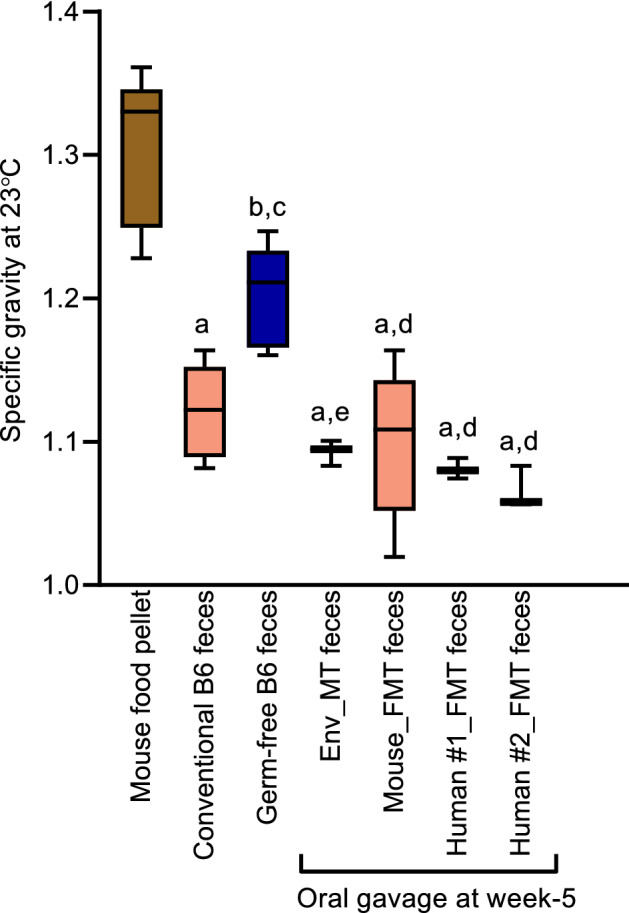
Specific gravities of feces. Specific gravity of the mouse food pellet, germ-free and gut-colonized (conventional, Env MT, mouse FMT and human FMT) mice fecal samples measured using pycnometer at 23 °C. Ordinary one-way ANOVA was performed for comparisons between the samples. ^a^*p *< 0.0001 versus Mouse food pellet, ^b^*p *< 0.01 versus Mouse food pellet, ^c^*p *< 0.05 versus Conventional B6 feces, ^d^*p *< 0.01 versus Germ-free B6 feces and ^e^*p *< 0.05 versus Germ-free B6 feces.

Next, we investigated the specific gravity of standard chow pellets and fecal pellets from all 5 groups of mice. Specific gravity was measured using a pycnometer as previously described^1^; *see* Methods) (Fig. 5). We observed that food pellets had the highest specific gravity of 1.304 and their fecal counterpart in germ-free mice had 8% lower specific gravity. The fecal pellets in all animals had a specific gravity higher than that of water. Interestingly, the specific gravity of feces were mostly comparable among all groups of gut-colonized mice namely conventional, Env MT, mouse FMT, and human FMT, but were significantly lower than germ-free mice. Our findings indicate that the microbial flora negatively impacts mouse fecal specific gravity, raising an interesting question on their differences in buoyancy.

### Levô in fimo test distinguish feces in germ-free versus gut-colonized mice

**Figure 6:**
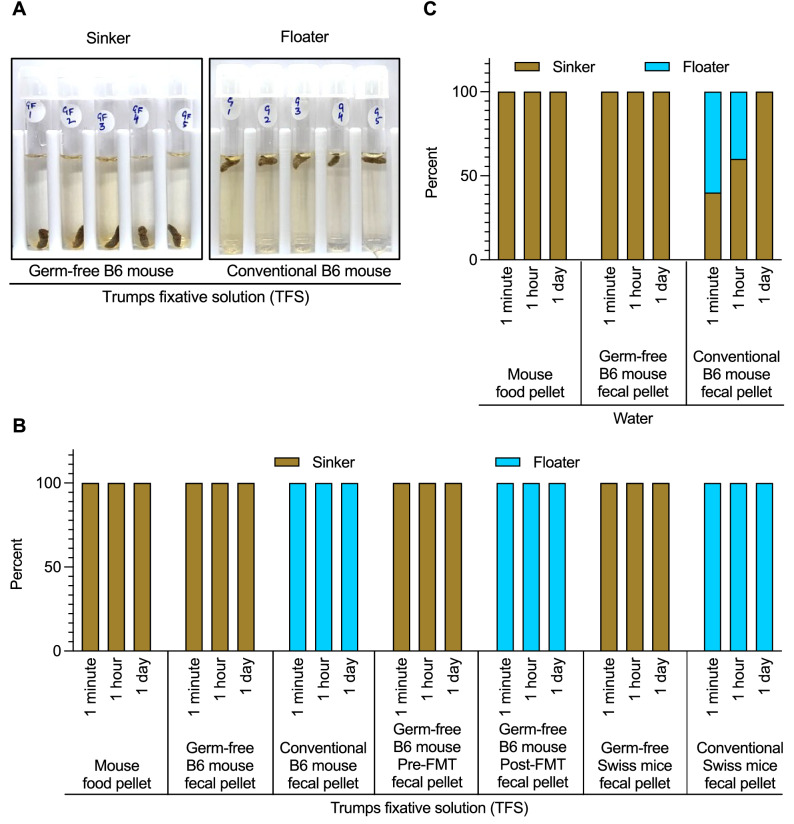
Levô in fimo test. (**A**) Representative levô in fimo test (LIFT) results of fecal pellets showing ‘sinkers’ and ‘floaters’ from germ-free and conventional in TFS. Floating and sinking feces indicates positive and negative LIFT results respectively. (**B**) LIFT efficacy over 24-h time period for different fecal pellets in water and (**C**) TFS for fecal pellets from germ-free and various gut-colonized mice. Bar plot summarizing LIFT results are presented. A minimum of 5 individual fecal pellets were tested per group of 5 mice each.

Herein, we determined the buoyancy of feces obtained from germ-free, conventional, and mouse FMT B6 mice in Trump’s fixative phosphate-buffered solution (TFS) and water. We observed that 100% of the fecal samples obtained from germ-free mice sank immediately (< 1 min) to the bottom of the tube in TFS, while 100% from conventional floated (Fig. 6A). We next tested the floatation of feces from germ-free mice before and after mouse FMT. In TFS, gut colonization of microbiota led to floatation of all fecal pellets tested (Fig. 6B). To determine if this is a general phenomenon, we tested the buoyancy properties of germ-free and conventional mice of another strain, namely Swiss Webster. Identical results were obtained in this strain of mice as well (Fig. 6B). The floating feces, irrespective of strain, did not sink, and vice versa was true during the observation periods of 1 min, 1 h and 1 day in TFS (Fig. 6B). Notably, nearly half of fecal samples from conventional mice sank when the floatation test was performed in water (or PBS, data not shown), indicating that the stringency to discriminate feces between germ-free versus gut-colonized mice was specific to TFS (Fig. 6C). The observation that certain fecal pellets with comparable microbiota densities sink in water suggests that the mere presence of microbiota may not be sufficient in promoting buoyancy. In fact, nearly 85% of human feces are known to sink in water^1^. The data from the floatation test suggests that lack of microbiota causes the feces to sink in TFS and gut colonizers confer the ability to float in TFS. The above-described method of determination of the floatation status of feces will be referred to hereafter as the levô in fimo test (also abbreviated as LIFT).

### Levô in fimo test tracks gut microbial reconstitution and genesis of fecal floatation

**Figure 7:**
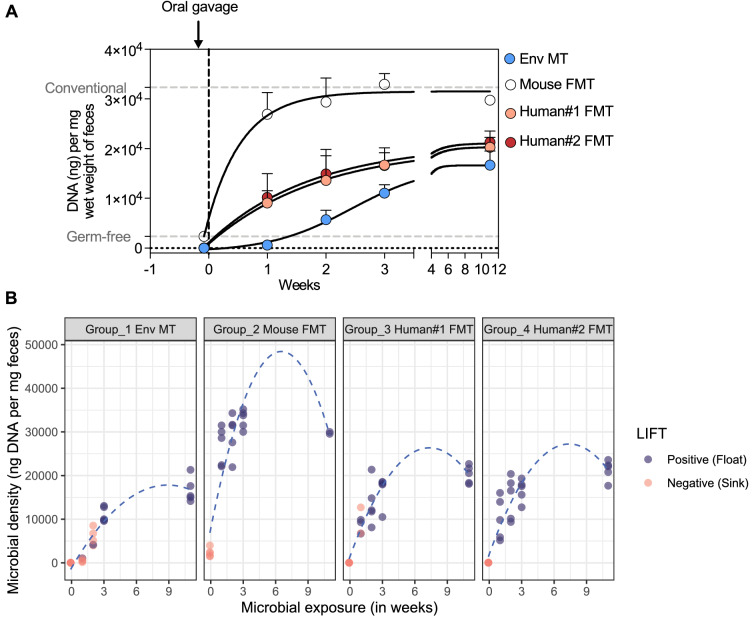
Levô in fimo test and gut microbial colonization kinetics. (**A**) Gut-colonization kinetics in Env MT, mouse FMT, human FMT#1, and human FMT#2 groups of mice. (**C**) Relationship between fecal microbiota density and fecal floatation instantly tested using LIFT in TFS. n = 5 fecal samples per group of 5 mice each.

Next, we performed simultaneous analysis of microbiota density and LIFT to demonstrate the utility of LIFT to understand early gut microbial reconstitution kinetics in germ-free mice (Supplementary Fig. 1A). We collected feces from germ-free mice before intragastric gavage and then weekly after intragastric gavage. Each fecal sample was split into two halves. One half was used for LIFT and the other for total fecal DNA measurement (an indicator of microbiota density) (Supplementary Fig. 1B). Prior to microbial transmission, all fecal samples tested negative (i.e., sank) in LIFT, and the results correlated with the lack of fecal microbiota densities (Fig. 7A–B). In the mouse FMT group, microbiota densities sharply increased and reached a steady state by 3 weeks and displayed levels similar to those of conventional mice (Fig. 7A). In this group, 100% of fecal pellets from post-intragastric gavage timepoints tested positive (i.e., floated) in LIFT (Fig. 7B). Notably, microbiota densities at 1-week post-intragastric gavage reached above 20 µg per mg wet feces (i.e., 2/3rd of steady-state levels) in this group.

Not surprisingly, our fecal analysis shows that both human FMT and Env MT mice had inferior gut colonization kinetics compared to that of mouse FMT mice, yet 100% tested positive (i.e., floated) in LIFT at 3 weeks or later post-intragastric gavage (Fig. 7B; Supplementary Fig. 2). At 2 weeks post-intragastric gavage, all fecal pellets from human FMT mice and 20% from Env MT mice tested positive (i.e., floated) in LIFT and the average fecal microbiota densities were nearly half in Env MT mice (5.7 µg per mg of wet feces) compared to human FMT (13.6 µg per mg of wet feces for Human FMT1 and 15.3 µg per mg of wet feces for Human FMT2) (Fig. 7B). A week post-intragastric gavage, although average fecal microbiota densities were similar, 100% of fecal pellets from human#1 FMT and 60% from human#2 FMT tested positive in LIFT (Fig. 7B). Discordance in LIFT results at early timepoints in feces with similar microbiota densities indicated that not all microbial species equally contribute towards fecal floatation.

### Metagenomic analysis identifies Bacteroides ovatus as a major gasogenic gut colonizer

Gaseous products of microbial fermentation in the gut influence the buoyancy properties of feces^1^. We searched the literature for microorganisms known or predicted to be gasogenic (Supplementary Table 1). These included methanogenic, H2-producing, ammoniagenic, sulfidogenic, denitrifying (NO-producing), acetogenic, and butyrogenic bacterial species. These gases are associated with flatulence in humans^7,15^. In fact, odorless methane is known to significantly contribute towards human fecal floatation^1^. We analyzed the metagenomes of feces from two different cages of 8-week-old conventional mice (conventional#1 and #2), four cages of age-matched 3-weeks post-fecal transplanted gnotobiotic mice (mouse FMT#1 to #4) and feces from donor B6 mice.We obtained a mean read depth of ~ 15 M reads/sample and following adapter trimming, quality filtration steps, fully optimal gapped DNA sequences were aligned to a curated reference database for taxonomic assignments.

**Figure 8:**
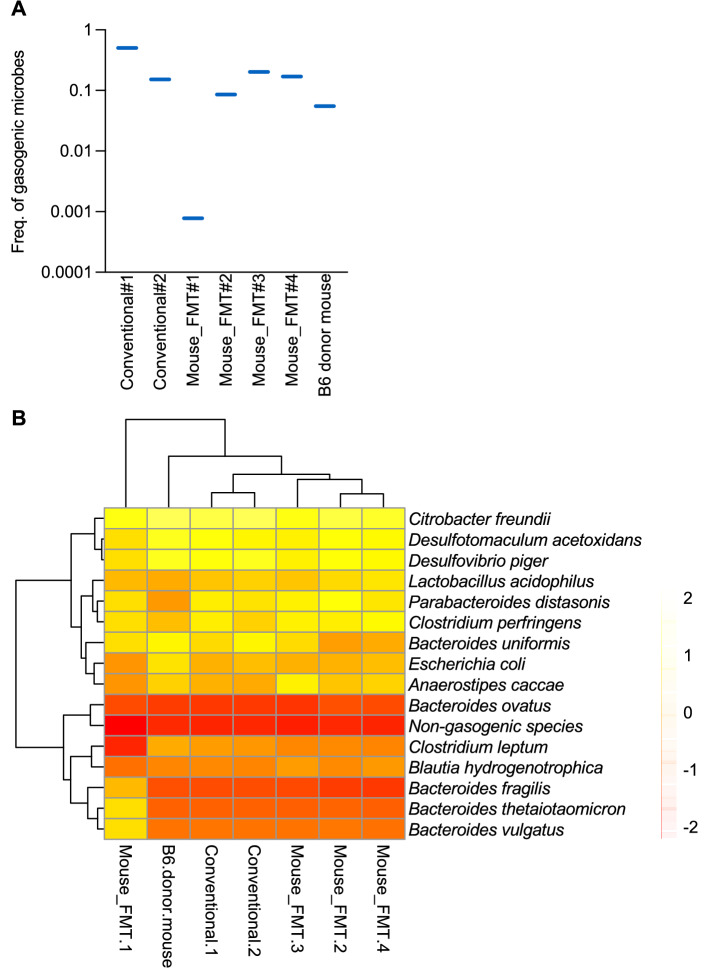
Metagenomic analysis of gasogenic microbial species. (**A**) Combined frequency of gasogenic gut microorganisms in the fecal samples of 8-week-old conventional mice and age-matched gnotobiotic mice following 3 weeks post-mouse FMT. Each feces were collected from a different cage. (**B**) Cluster analysis and heatmap of individual gut microorganisms in the fecal samples of 8-week-old conventional mice and age-matched gnotobiotic mice following 3 weeks post-mouse FMT.

Different frequencies of gasogenic species were identified in mouse fecal metagenomes (Supplementary Table 2). The frequency of total gasogenic gut bacteria in 8 week old mice ranged from ~ 0.000777 to 0.503742 (mean ± SEM; ~ 0.166728 ± 0.164120) (Fig. 8A) depicting a high-degree of inter-mouse variations in gut colonization of these microorganisms. Fecal specimens from mouse FMT#1 and conventional#1 had the lowest and highest proportion of gasogenic microorganisms, respectively (Fig. 8A).

Species level analysis of gasogenic microorganisms identified 13 species from 11 genera, including *Bacteroides ovatus, Bacteroides fragilis, Bacteroides uniformis, Bacteroides thetaiotaomicron, Clostridium leptum, Clostridium perfringens, Escherichia coli, Blautia hydrogenotrophica, Parabacteriodes distasonis, Desulfotomaculum acetoxidans, Desulfovibrio piger, Anaerostipes caccae, Lactobacillus acidophilus* and *Citrobacter ferundii* (Fig. 8B). *Bacteroides* genus was overrepresented with the presence of five species namely *B. ovatus, B. uniformis, B. vulgatus, B. thetaiotaomicron* and *B. fragilis*. The frequency of total gasogenic bacteria in feces ranged approximately from 0.0004 to 0.5 (mean ± SEM; ~ 0.163 ± 0.0617). Notably, ~ 50% fecal bacteria in conventional#1 and ~ 0.0004 in mouse FMT#1 were gasogenic demonstrating heterogeneity in gasogenic gut microbial composition. Species-level analysis identified *B. ovatus* (frequency ranged from 0.0001 to 0.481) and *B. fragilis* (frequency ranged from 0.00009 to 0.0157*)* as two abundant gasogenic species (Fig. 8B; Supplementary Table 2). In conventional#1, *B. ovatus* alone contributed to 96.5% of gasogenic microorganisms suggesting that this species may be a major contributor to fecal floatation. *B. ovatus* was most abundant gasogenic species except mouse FMT#1 where *Clostridium leptum*, a species less represented in feces of inflammatory bowel disease patients^16^, was more abundant than *B. ovatus* (~ 0.000318 vs. ~ 0.000149) (Fig. 8B; Supplementary Table 2). *B. ovatus* and *B. uniformis* have been implicated in gas production through the fermentation of carbohydrates and gas evacuation in patients with flatulence, suggesting a conserved microbial mechanism of fecal gasogenesis between mice and humans^17^. Together, our study strongly implicates gut-colonizing microorganisms with biomass transformation leading to fecal floatation properties in mice.

## Discussion

Our serendipitous finding of ‘sinker’ and ‘floater’ feces in TFS in germ-free and gut-colonized mice, respectively, led to the question of whether gut colonizers were fundamentally linked to the genesis of fecal floatation phenomenon. By introducing microorganisms into the gut of germ-free mice, we have conclusively demonstrated that gut colonization of microbiota is a pre-requisite for feces to float.

**Figure 9:**
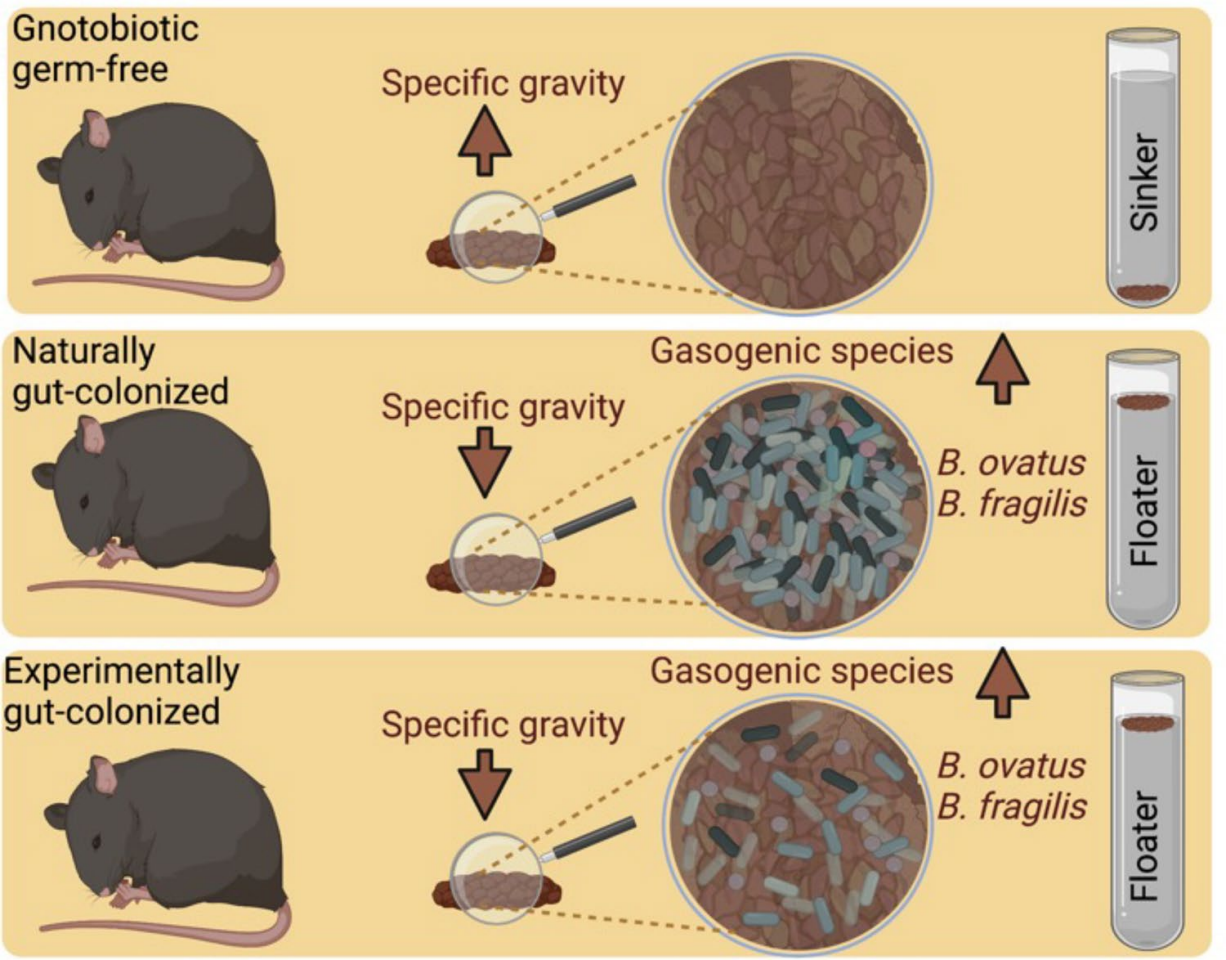
Illustration showing the role of gut microbial influence on fecal floatation in mice. The illustration was created using in BioRender.com.

Gut colonization and microbial transformation of food are causally linked to a reduction in fecal specific gravity and a corresponding increase in buoyancy. The LIFT in TFS instantaneously distinguishes feces from germ-free versus gut-colonized mice (Fig. 9). To distinguish the fecal floatation described here from that in the literature which describes a fecal parasite swim test^18^, we refer to our method as LIFT. The latin term *in fimo* was recently introduced in scientific parlance to describe feces examined experimentally^19^. The latin word levô means ‘to levitate or float’. Therefore, we consider the levô in fimo test (abbreviated as LIFT) to be an appropriate name for the test to measure the intrinsic floatation property of feces.

Presently, there is no standardized method to delineate gasogenic versus non-gasogenic gut microbial species. In vitro microbial anaerobic culture systems do not capture gut physiology and the complex interactions of gut microbiota with food and host systems^20^. In gnotobiotic mouse models, LIFT combined with microbiome analysis could further delineate microorganisms as gasogenic or non-gasogenic under various defined health, diet, and lifestyle conditions. To our knowledge, LIFT, validated here using two different strains of germ-free and gut-colonized mice, is the first method to discriminate feces based on floatation property. LIFT is compatible with feces obtained fresh, during cage changes, or fresh frozen (data not shown).

The nature of microbial transformation and attendant floatation property in the presence or absence of gut colonizers was explored in the present study. In general, feces from gut-colonized mice floated in LIFT in TFS. The examples of microbially-rich fecal pellet sinking in TFS during early stages of gut colonization or more commonly in water highlight that the mere presence of microorganisms may not be sufficient to float. We suspect that in addition to the presence of microorganisms, an enrichment of gasogenic microorganisms may be essential in aiding fecal floatation. A study by Levitt and Duane using floating and sinking feces from healthy volunteers concluded that human feces float primarily due to increased gas content in them^1^. Our study showed enrichment, albeit at variable frequency, of gasogenic microbial flora in naturally and experimentally gut-colonized mice. Together, these studies suggest gasogenic microorganisms as a factor influencing fecal buoyancy across mammals.

The gut of a healthy mice is reported to be composed of 37 core genera of bacteria that influence host metabolism and the immune system^21^. *Bacteroides* is identified as one of the predominant core genera in human and mouse fecal samples^21,22^. The members of the genus *Bacteroides,* including *B. ovatus* and *B. fragilis,* produce gases (H_2_ and CO_2_) primarily through the metabolism of carbohydrates in the gut. Subsequently, the free hydrogen is utilized by hydrogenotrophic bacteria, including methanogens and sulfate reducing bacteria to produce CH_4_ and H_2_S^23–25^. The metagenomic analysis of mouse fecal samples identified four gasogenic species of genus *Bacteroides*. In fact, we identified *Bacteroides ovatus* to be the most enriched species in our analysis which has been positively correlated with flatulence and anal gas evacuation in human patients^17^. Further, we also identified *Bacteroides fragilis* which is known to produce hydrogen gas in the gut^26^. However, we observed heterogenous and lower proportions of hydrogenotrophic microorganisms relative to *Bacteroides*. Nonetheless, our study suggests that food transformation to microbial biomass is necessary but insufficient to cause feces to float, and an increase in the abundance of gasogenic microorganisms may help overcome this insufficiency. Further investigation is required to identify various gasogenic microbial communities and their minimum frequencies to overcome necessary threshold to cause feces to float in mice and humans. Also pending investigation is the outstanding question in gastroenterology; whether floating stools are associated with specific functional bowel disorders and are potential gasogenic subsets of microbiota linked to fecal floatation^2,27^.

The study offers other insights pertaining to the nature of gut colonizers that influence the floatation of feces. The significant negative correlation reported here between FACS-based gut microbiota density and fecal UFP could be of interest as it will enable investigation into the efficiency of food-to-microbial biomass transformation (referred to as microbial transformation) by specific gut colonizers. Gut colonizers are involved in microbial transformation of otherwise unavailable oligosaccharides and in synthesis of essential nutrients from dietary fiber^28–30^. The presence of fermentable substrates causes a corresponding increase in the microbiota densities in the feces^31^. In fact, the lack of microbial transformation has been shown to cause nutritional and digestive deficiencies in germ-free mice^32^.

Our findings help understand the fundamental role of microorganisms in food biotransformation, fecal buoyancy, and flatulence in regulating human gut health^33^, on earth and outer space. The approaches described here will pave way to conduct research to understand the structure–function of FACS-detectable fecal UFP and their relationship to gut health, and to explore the effects of complex diet, antibiotics, pathological conditions such as colitis, donor fecal characteristics (complex or defined) on gut-colonized gasogenic bacteria and attendant fecal floatation property. Such information may also guide careful screening of fecal donors for FMT in clinic.

In conclusion, our study reports causal link between gut microbial colonization and fecal floatation in mice. The findings may be relevant in improving our understanding of food microbial biotransformation and gut microbial regulators of fecal floatation in human health and disease.

## Methods

### Germ-free and conventional mice

Female 6–8-week-old conventional C57BL/6NTac (B6) mice were obtained from Taconic Biosciences and maintained in standard mouse facility. Mayo Clinic Germ-free Facility bred and maintained germ-free B6 mice (originally obtained from Taconic Biosciences) in sterile isolators (Class Biologically Clean, Ltd). Age-matched female germ-free B6 mice were used in this study. Conventional mice were fed with PicoLab mouse diet 20 5058. Germ-free mice were fed an autoclaved standard diet (LabDiet, 5K67). All procedures were reviewed and approved by Mayo Clinic Institutional Animal Care and Use Committee. This study was carried out in compliance with the ARRIVE guidelines.

### Fecal sample collection

Excreted mouse feces were collected from the cages of germ-free and gut-colonized mice once a week during cage changes. The human feces were collected, stored, and manipulated after receiving informed consent from the subjects under the approved procedures of the Mayo Clinic’s Institutional Review Board, and all methods were performed in accordance with the relevant guidelines and regulations. To control for process variables and to obtain consistent results, common methods for human and mouse fecal sample collection, storage and processing were used. Fecal pellets were freshly collected and frozen at – 80 °C. For the levô in fimo test, all fecal and food pellets were processed in the research laboratory at standard room temperature of 23 °C and 1 atmosphere pressure.

### Fecal microbiota transplantation (FMT) to conventionalize germ-free mice

A fecal pellet of freshly collected and frozen from cages of conventional B6 mice was used to gavage 2 mice. In brief, a fecal pellet was homogenized in 750 µl pre-reduced PBS in an anaerobic chamber (Coy Laboratory Products) and 300 µl of fecal suspension was loaded into 1 ml syringes attached with gavage needles. Subsequently, the mice were restrained by hand, and the gavage needle was inserted through the esophagus into the stomach, and fecal suspension was delivered by depressing the syringe plunger. The needle was removed, and the mouse was placed back into the cage. All the mice received fecal preparation from single donor cage. For human FMT, the frozen feces samples were mixed with equal volume (50/50) pre-reduced PBS in an anerobic chamber and 300 µl of fecal suspension was gavaged into a mouse. Each mouse of PBS group was gavaged with 300 µl of sterile pre-reduced PBS.

### Monitoring germ-free status using anaerobic and aerobic microbial culture method

Fecal pellets were placed into a clean culture hood and added to two sets of tubes with either Brain- Heart Infusion (BHI) broth medium for the growth of aerobic bacteria, nutrient broth medium fastidious and non-fastidious microorganisms or Sabouraud Dextrose (SD) broth medium for the cultivation of yeast and molds^34^. One set of tubes with each broth was placed into the incubator inside of the anaerobic chamber. The other set with each broth was placed into a standard aerobic incubator. Samples were checked daily for a week for microbial growth. The isolator and mice were referred to as ‘germ-free’ if all tubes tested by this method remained clear during this period.

### Scanning electron microscopy (SEM)

The fecal samples were fixed at 4 °C for a day in Trumps fixative, 4% formaldehyde + 1% glutaraldehyde in a phosphate buffer^35^. Samples were then washed in phosphate buffer, rinsed in water, and dehydrated through a graded series of ethanols (10%-30%-50%-70%-90%-95%-100% × 2). Then critical point dried (EMS) using carbon dioxide, mounted on an aluminum stub using conductive carbon SEM tabs (EMS), and sputter-coated with gold–palladium for 150 s. Finally, they were imaged in a Hitachi S-4700 cold field emission scanning electron microscope SEM (paired upper and lower secondary electron (SE) detectors) at 5 kV accelerating voltage. All micrographs were acquired as TIFF images.

### Fecal DNA isolation

Fecal DNA was extracted using MasterPure complete DNA and RNA purification kit (MC85200) following manufacturer’s instructions with some modifications. In brief, 10 mg of fecal pellet was transferred to a tube with 300 µl tissue and lysis solution containing Proteinase K and homogenized using BioMasher II Closed System Disposable Micro Tissue Homogenizer. Samples were then incubated at 65 °C for 1 h with agitation in Eppendorf Thermomixer C. The samples were cooled, centrifuged and the supernatant was transferred into a fresh tube and treated with RNase A at 37 °C for 30 min. Subsequently, MPC protein precipitation solution was added to the samples, centrifuged and DNA was precipitated from the supernatant using isopropanol. The precipitated DNA was washed twice with 70% ethanol and resuspended in TE buffer. The yield of extracted DNA was estimated using Implen NanoPhotometer NP80. For engraftment kinetics, fecal DNA was extracted at different time points (2 h prior to fecal transplant and then 1, 2 and 3 weeks post-fecal transplant) from excreted feces obtained during cage changes.

### Microbiome analysis by PCR

Microbiome analysis was performed with 500 ng of fecal DNA using 5 PRIME HotMasterMix and Universal (forward 5’-AGA GTT TGA TCC TGG CTC AG and reverse 5’-GAC GGG CGG TGW GTR CA) and Turicibacter (forward 5’-GCG CGC AGG TGG TTA ATT AAG TCT and reverse 5’-TCA GTG TCA GTT GCA GAC CAG GAA) primers as described elsewhere^36,37^. The following PCR cycling conditions were used for amplification: initial denaturation of 94 °C for 2 min, followed by 30 cycles of 94 °C for 1 min, 55 °C for 3 min, 72 °C for 3 min with a final extension of 72 °C for 20 min using a Gene Touch thermal cycler (Bulldog Bio, Inc).

### Quantitative flow cytometry to measure fecal microbiota density

Each fecal pellet was weighed, and microorganisms were extracted in sterile PBS as described previously with following modification^38^. Briefly, a fecal slurry was prepared in sterile PBS and the fecal debris were removed by centrifugation at 300 g for 5 min at 4 °C. The supernatant was diluted in sterile PBS and centrifuged at 15000 g for 5 min at 4 °C and particles carrying microorganisms were pelleted. The pellet was then resuspended in 4% paraformaldehyde and fixed for 1 h at room temperature and centrifuged at 15000 g for 5 min at 4 °C. The pellets were washed twice in PBS. The particles were finally resuspended in PBS and spiked-in with CountBright Absolute Counting Beads, for flow cytometry (Invitrogen) and filtered through 40 µm strainer. Microorganisms and UFP were quantified using BD FACS Melody Cell Sorter. The data was analyzed using FlowJo v10.8.1.

### Conventional thermogravimetric analysis (TGA)

Thermalgravimetric combustion analysis was performed on a Thermogravimetric Analyzer- Discovery HP TGA, TA instruments (Delaware, USA). Freshly collected single fecal pellets (~ 15 mg) from conventional, germ-free, and mouse fecal transplanted B6 mice were analyzed in 4 replicates by heating dynamically from 30 to 700 °C in platinum crucibles (70 µl) at a heating rate of 10 °C /minute under Nitrogen atmosphere for pyrolysis at 30 ml/minute gas flow rate. The weight loss percentage, peak combustion temperature, and combustion char residue characteristics were obtained from thermogravimetric analysis and first derivative (dw/dt) weight loss curves were evaluated quantitatively using Trios v5.1.0.46403 Software (TA instruments).

### Fecal specific gravity measurement using pycnometer

A pycnometer was allowed to stabilize at room temperature and weighed with and without distilled water completely filling the pycnometer up to the capillary of the stopper. The weights were noted, and the pycnometer was dried completely. Next, the mouse fecal pellets (n = 13 to 18) were added to the pycnometer stabilized at room temperature of 23 °C and was weighed along with the stopper. The pycnometer was then filled completely with distilled water up to brim of capillary stopper and gently tapped few times to remove any air bubbles. The surface of pycnometer was wiped dry and weighed again. The weight readings were then used to calculate the specific gravity of the fecal samples as described previously^39^.

### Levô in fimo test (LIFT)

Individual or half fecal pellet from germ-free or gut-colonized mice were dropped into 3 ml of a solution (either TFS, pH 7.2 or water purified using a Millipore Milli-Q Lab Water System, pH 7.0) in 5 ml clear tubes. The samples were observed for floatation at 1 min, 1 h and 1 day period and photographed. Test result was noted as positive if feces floated and negative if feces sank to the bottom of the clear tube.

### Fecal metagenomics analysis

Whole-genome shotgun sequencing was performed using 200 ng of fecal DNA isolated from the cages of 8-week-old gut-colonized (conventional and mouse FMT) mice and the single B6 donor mouse on NovaSeq 6000 system (Illumina). The samples were sequenced with a 150-bp read length for paired-end sequences with a targeted sequencing depth of greater than 2 M reads/sample. Reads were subjected to adapter trimming, quality trimming, and entropy filtering via BBDuk (v. 38.69) (https://sourceforge.net/projects/bbmap/). The reads were then screened for human and mouse genomic content using BioBloom Tools (v 2.3.2)^40^ with hg38 and GRCm49 used as references, respectively. The screen reads were further processed with SHI7 (v 1.0.0)^41^. Taxonomic assignment was performed on the quality-controlled reads using SHOGUN (v. 1.0.8)^42^ and RefSeq82 as the reference database. Gasogenic bacteria were identified via literature search and selected from the resulting taxonomic tables. Clustering and heat map of gasogenic microorganisms were generated using the “pheatmap” package in R (version 1.0.12, https://cran.r-project.org/web/packages/pheatmap). All the analyzed files are available on the Sequence Read Archive (SRA) under the BioProject accession number PRJNA802607.

### Preprint

A portion of this manuscript was published as a preprint^43^.

### Ethics approval

All procedures were approved by Mayo Clinic Institutional Animal Care and Use Committee and all methods were performed in accordance with the relevant guidelines and regulations. This study was carried out in compliance with the ARRIVE guidelines. The human feces were collected, stored and manipulated after receiving informed consent from the subjects under the approved procedures of the Mayo Clinic Institutional Review Board.

## Supplementary Information

Supplementary Information.

## Data Availability

All data generated or analyzed during this study are included in this published article (and its supplementary information files). Fecal metagenomic analysis files are available on the Sequence Read Archive (SRA) under the BioProject accession number PRJNA802607.

## Abbreviations

FMT: Fecal microbiota transplant
LIFT: Levô in fimo test
PCR: Polymerase chain reaction
EM: Electron microscope
DNA: Deoxyribonucleic acid
FACS: Fluorescence-activated cell sorting
UFP: Undigested food particles
g: Gram
mg: Milligram
µg: Microgram
TGA: Thermogravimetric analysis
TFS: Trump’s fixative phosphate-buffered solution
PBS: Phosphate buffered saline
SE: Secondary electron
NO: Nitric oxide
H_2_: Hydrogen
CO_2_: Carbon dioxide
H_2_S: Hydrogen sulphide
CH_4_: Methane
SD: Standard deviation
SEM: Standard error of the mean
ANOVA: Analysis of variance
TIFF: Tag image file format
