# Supplementary material for: Genesis of fecal floatation is causally linked to gut microbial colonization in mice
Source: Sci Rep. 2022 Oct 27;12:18109. doi: 10.1038/s41598-022-22626-x (PMC9613883; doi:10.1038/s41598-022-22626-x)
Supplement: Supplementary file 1 — Supplementary Information. [file 41598_2022_22626_MOESM1_ESM.pdf]

Supplementary information

Supplementary Figure 1. Split-feces assay. A) Schematics showing gnotobiotic germ-free B6 mice gut colonization using different microbial exposures (Env MT, mouse FMT, human FMT#1, and human FMT#2) and split-fecal assay to test microbial density and floatation (n=5 fecal samples per group of 5 mice each). The illustration was created using in BioRender.com.

Supplementary Figure 2. Efficacy of LIFT in TFS over 24-hour time-period in different groups of gut-colonized mice (n=5 fecal samples per group of 5 mice each).

Supplementary Table 1. List of gasogenic commensal gut microorganisms.

Supplementary Table 2. Observed frequencies of gasogenic gut microorganisms in feces.

Supplementary Figure 1

A

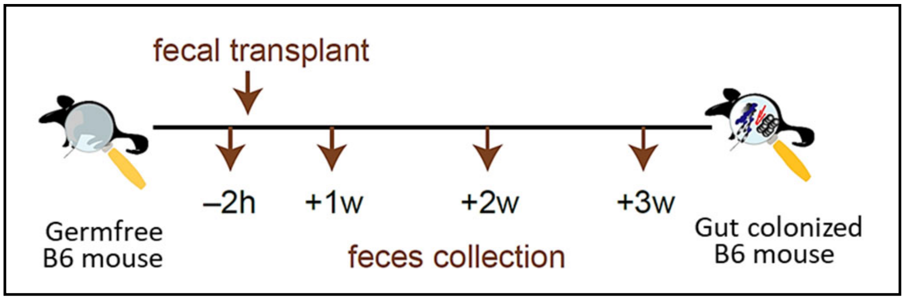

B

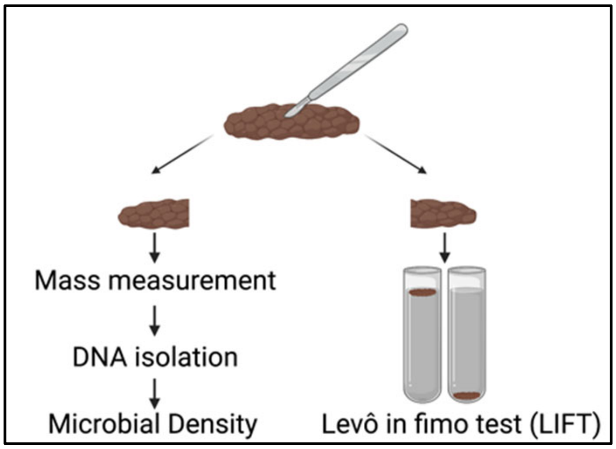

Supplementary Figure 2

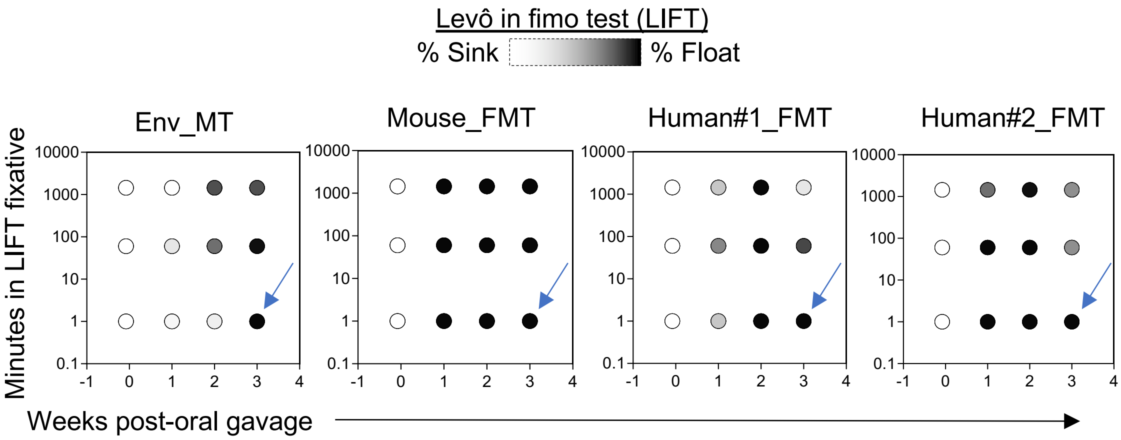

Supplementary Table 1. List of gasogenic commensal gut microorganisms.

List of known or predicted gasogenic gut microorganisms
Anaerostipes caccae
Atopobium parvulum
Bacillus anthracis
Bacillus subtilis
Bacteroides fragilis
Bacteroides ovatus
Bacteroides thetaiotaomicron
Bacteroides uniformis
Bacteroides vulgates
Bifidobacterium bifidus
Bifidobacterium breve
Bifidobacterium infantis
Bifidobacterium pseudocatenulatum
Bilophila wadsworthia
Blautia coccoides
Blautia hydrogenotrophica
Christensenella minuta
Citrobacter freundii
Clostridium histolyticum
Clostridium leptum
Clostridium perfringens
Clostridium ramosum
Deinococcus radiodurans
Desulfitobacterium hafniense
Desulfobacterium autotrophicum
Desulfobulbus propionicus
Desulfococcus multivorans
Desulfotomaculum acetoxidans
Desulfovibrio desulfuricans
Desulfovibrio farfieldensis
Desulfovibrio piger
Desulfovibrio vulgaris
Edwardsiella tarda
Enterobacter aerogenes
Enterobacter cloacae
Escherichia coli
Eubacterium hallii
Fusobacterium nucleatum
Lactobacillus acidophilus
Lactobacillus rhamnosus
Lactobacillus shirota
Methaninobrevibacter smithii
Methanobrevibacter ruminantium
Methanococcus maripaludis
Methanosphaera stadtmanae
Parabacteroides distasonis
Proteus vulgaris
Ruminococcus hydrogenotrophicus
Salmonella typhimurium
Veillonella ratti

Supplementary Table 2. Observed frequencies of gasogenic gut microorganisms in feces.

Name	Conventional#1	Conventional#2	Mouse_FMT#1	Mouse_FMT#2	Mouse_FMT#3	Mouse_FMT#4	Conventional#3 (donor)
Bacteroides ovatus	0.481675797	0.144329671	0.000149785	0.022491187	0.191628505	0.023910416	0.052449192
Bacteroides fragilis	0.015744498	0.005363504	9.36154E-06	0.04920094	0.006631645	0.127485169	0.001733877
Bacteroides thetaiotaomicron	0.004700832	0.001465757	0	0.0089953	0.001837869	0.011225808	0.000416034
Bacteroides vulgatus	0.001357653	0.000463063	0	0.004459459	0.000673885	0.004932131	0.000262126
Clostridium leptum	8.05387E-05	6.92771E-05	0.000318292	9.40071E-05	0.000329285	0.000694666	4.32868E-05
Blautia hydrogenotrophica	0.000131492	0.000102093	0.000140423	9.40071E-05	0.000153156	0.00012504	6.01206E-05
Escherichia coli	1.47928E-05	2.1877E-05	7.48923E-05	2.93772E-05	0.000145498	6.94666E-05	9.61929E-06
Anaerostipes caccae	1.47928E-05	4.74001E-05	7.48923E-05	2.35018E-05	0	4.168E-05	1.44289E-05
Lactobacillus acidophilus	9.86188E-06	1.82308E-05	9.36154E-06	1.17509E-05	3.82889E-05	2.77867E-05	4.32868E-05
Bacteroides uniformis	8.21824E-06	3.64616E-06	0	5.2879E-05	3.06312E-05	9.72533E-05	2.40482E-06
Parabacteroides distasonis	1.64365E-06	7.29232E-06	0	0	0	2.77867E-05	4.80965E-05
Clostridium perfringens	1.64365E-06	1.82308E-05	0	5.87544E-06	0	0	1.92386E-05
Desulfotomaculum acetoxidans	0	3.64616E-06	0	0	0	0	0
Citrobacter freundii	0	0	0	0	0	0	0
Desulfovibrio piger	0	0	0	0	0	0	0
Total Gasogenic	0.503741763	0.151913688	0.000777008	0.085458284	0.201468764	0.168637204	0.055101712
Other non-gasogenic	0.496258237	0.848086312	0.999222992	0.914541716	0.798531236	0.831362796	0.944898288

Note: *C. freundii* and *D. piger* are occasionally detected in minor frequency in fecal samples from older B6 mice (data not shown) but not in these 8 week old.
